# Supplementary material for: The role of scenic context on upright face preference in infancy
Source: PLoS One. 2023 Jul 13;18(7):e0288253. doi: 10.1371/journal.pone.0288253 (PMC10343060; doi:10.1371/journal.pone.0288253)
Supplement: Attachment — Submitted filename: ResponseToEditor_R2.docx [file pone.0288253.s004.docx]

Thank you for submitting your manuscript to PLOS ONE. After careful consideration, we are please to see that all reviewers' comments have been successfully addressed. Your efforts in improving the paper are highly appreciated.

However, before we can accept your paper for publication, we need you to report exact p-values for all values greater than or equal to 0.001 as per journal guidelines. Accurate reporting of p-values is essential for ensuring the reproducibility and validity of research findings. Therefore, I kindly ask you to submit a revised version with the exact p-values.

*Response- According to your suggestion, we have revised the p-values to report exact values for all greater than or equal to 0.001.*
